# Understanding Bidirectional Reflectance and Transmission for Space Applications

**DOI:** 10.6028/jres.080A.058

**Published:** 1976-08-01

**Authors:** John B. Schutt

**Affiliations:** Goddard Space Flight Center, Greenbelt, Maryland 20771

**Keywords:** Bidirectional radiometry, canopies, diffuse reflectance, diffuse transmission, reflectance standards, scattering surfaces

## Abstract

Applications for optical diffusers in space projects are presented which include the functions of reflection, transmittance, and collection. These modes encompass such diverse uses as temperature regulation and ozone concentration monitors. Discussed is the cooperative aspect of diffuse reflectance and environmental stability. Magnesium oxide, sodium chloride and barium sulphate are evaluated in some detail. The importance of scene scattering behavior to modeling the earth’s radiation budget and in determining thermal inertias of the earth’s surface are discussed, because solar albedo serves as the weighting function in the solar input irradiance. Finally, work in the area of canopy reflectance modeling is reviewed with verification data included whenever available. Some knowledge of the bidirectional reflectance properties of vegetation is necessary for identification, acreage computations, and scene transference.

## I. Introduction

Uses of diffusely reflecting and transmitting materials in spacecraft design are numerous, so numerous in fact that they are taken for granted. They are taken for granted in the sense that the extent of their diffuseness often is judged by eyeballing. Perhaps this turns out to be the situation because the experimental procedure for determining diffuseness requires great care, and becomes tedious if the hemisphere is to be covered for wavelengths comprising the solar spectrum, 250 to 2500 nm. On the other hand, it is customary to carry out bidirectional reflectance measurements on diffusers applied to the surfaces of integrating spheres, but normally in the principal plane only.

The nature of the application of diffusers usually puts them in a pivotal role. For example, in the control of satellite temperatures, mirror effects are eliminated whereby solar inputs exceeding one solar constant can occur for coupled surfaces. In the development of spacecraft paints, it turned out that certain pigments, particularly zinc oxide, can be processed to have greater environmental stability than any type of binder. Inorganic and silicone binders have intermediate stabilities, but can be well masked because they can accept high pigment concentrations, so high in fact that the resultant coating becomes a diffuser. Under these circumstances there results classes of paints which are surprisingly space stable [1].^1^ Another pivotal role is that of a collector. Reflectors are utilized in this manner to monitor ozone concentrations in the earth’s atmosphere. The collector utilized in the Backscatter Ultraviolet Experiment (BUV) [2, 3] flown on Nimbus 3 and 4 is a ground aluminum plate overcoated with an evaporated layer of aluminum. This configuration was selected after much screening because its long term environmental stability is excellent [4]. Although it has an easily detected specular quality, the instrumentation is sufficiently sensitive to enable detection of components from 250 to 300 nm which comprise the earth’s albedo and are representative of the ozone profile. To provide calibration of BUV, sounding rockets are launched from selected sites during overhead passage of the satellite. The pivotal element in this ozone monitoring experiment is a collector comprising a silica sphere coated internally with a transmission diffuser. Originally, smoked magnesium oxide was employed, but ease of application prompted a switch to barium sulphate bound with polyvinyl alcohol. In optimum configuration, however, Krueger [5] found that a black spot placed exterior to the sphere opposite to the exit neck could eliminate illumination fluctuations, thereby providing a more uniform flux to the detector.

Most recently, interest has developed in the reflective patterns of the earth and earth scenes. Nimbus F (currently in orbit) [6] has an experiment designed to monitor the earth’s energy balance. Formally known as the Earth Radiation Budget Experiment (ERB), this experiment has been designed to hopefully permit the calculation of energy balances to an accuracy approaching one percent. Broadly, the experiment views the sun as the satellite ascends from the South Pole. As it proceeds northward energies are measured from 0.3 to 50 *µ*m. At the same time the entire disk is viewed and synopsized. The goniometric dependence is taken in the principal plane of the sun and assumed to characterize the reflectance hemisphere. These data provide all the necessary input for calculating terrestrial energy balances. Planned for launch in the spring of 1978 is a scanner designed to measure reflected energy from 500 to 1100 *µ*m and characterize emitted energy in the 10.5 to 12.5 *µ*m band. The purpose of the Heat Capacity Mapping Mission (HCMM) [7] is to characterize the earth’s surface with respect to the mission spectroscopy. Because the spacecraft will pass over the same area at about 1:30 pm and 2:30 am, the most sophisticated application for its data will be for computations of thermal inertia. For a representative value of this quantity an energy balance must be established at the earth’s surface. Simplifying assumptions which enter modeling of the energy balance are:

1. that the scene is Lambertian,^2^
2. that the 500 to 1100 *µ*m band adequately estimates the solar albedo normally defined for energy balance purposes from 250 to 4000 *µ*m,
3. that the Planck function will peak in the 10.5 to 12.5 *µ*m interval permitting a reliable brightness temperature to be calculated,
4. meteorlogical approximations which involve the connective and advective heat transfer between the earth and atmosphere,
5. radiation contributions which result from exchanges between earth and atmosphere, earth and sensor and earth reflected sky radiation,
6. an atmospheric transfer function in order to correct sensed radiations to the earth’s surface.

If we are content with relative thermal inertias rather than representative values, certain of these approximations may be useful provided sufficient sensitivity is offered by such a modeling scheme to provide useful images. The simplest images offered by the HCMM are those obtained from mapping:

1. albedo
2. differential temperatures
3. a ratio of 1 to 2.

Of the three, the ratio of one to two offers the closest approximation to thermal inertia.

Finally, there are remote sensing requirements for crop signatures; these again may be representative or relative. But establishing a library of these data goes beyond spectral characterization alone and would entail knowledge of the diffuse versus specular quality of the reflectance as a function of sun and sensor positions. For example, viewing mature barley and wheat from the nadir position does not provide a basis for differentiation, however, as the view angle is increased, distinctions become increasingly apparent [8]. Otherwise, exclusive use must be made of differing spectral signatures occurring from phenological changes recorded in conjunction with individual crop calendars. These data provide bases for crop identification and acreage estimations.

In the following sections a summary of white diffusers is given. Subsequently, radiation transfer models employed in calculating the reflectances of crops are presented and verification data included whenever available.

## II. Summary of Information on Selected Diffusers

Of all the diffusers, magnesium oxide [9] generated and applied by burning magnesium under controlled conditions was probably the one most utilized until the development of the barium sulphate-polyvinyl alcohol system [10]. Disadvantages of magnesium oxide include:

1. its slow rate of deposition by virtue of the smoking process,
2. a thickness of 3 to 4 mm to provide capacity in the 0.7 to 2.4 *µ*m region,
3. a coating with poor adhesion and weak cohesion,
4. a required investment in time of three days to two weeks of continuous effort to coat an eight inch diameter integrating sphere,
5. a coating not accessible to repairing,
6. a system of electrostatically charged particles reactive with atmosphere moisture to form Brucite, Mg(OH)_2_, with an accompanying decrease in infrared and ultraviolet reflectances.

Under ideal conditions the usefulness of this coating may extend to one year.

A more rugged integrating sphere coating possessing optical properties comparable to magnesium oxide can be provided by sodium chloride. Initially developed by Kneisel [12] at the National Bureau of Standards, the system was modified by Stuart [13] to increase its ease of application and mechanical strength. Initially sodium chloride was dispersed in alcohol, but improvements were obtained by employing an admixture of toluene and 1, 3 butanedial or propylene glycol with the alcohol. The resultant coating is free of the usual water bands occurring in the vicinity of 1200, 1400, and 1900 nm. Furthermore, the system is easily repaired and dries under sufficient compressive stress to provide a coating able to support a substantial shock. The life of the coating is known to be in excess of two years. Its major disadvantage resides in its moisture sensitivity. Relative humidities close to 90 percent cause sagging. Optically, the coating commences to lose reflectance around 360 nm, but remains the best all around integrating sphere coating for determining the solar albedo of materials.

Ease of application, repair, and optical stability make the barium sulphate-polyvinyl alcohol coating the most useful system. It does have infrared absorptions due to the presence of water, but sources of energy in this spectral region have sufficient intensity to eliminate this as a potential disadvantage. Furthermore the system does not suffer from the near ultraviolet fall off characteristic of sodium chloride. Over the spectral region employed in solar albedo calculations, this system has been found to have a lifetime in excess of two years. However, Krueger [15] has shown that a substantial loss in reflectance can occur from about 250 to 200 nm within a period of three months. In an attempt to better utilize the high reflectance of virtually pure barium sulphate in the region of 200 to 250 nm for the express purpose of detecting Cerenkov radiation [16], it was found that polyvinyl alcohol could be replaced by potassium sulphate while maintaining the reflectance of the system to within 2 percent of that of the powder. The coating was monitored for optical degradation in this region over a period of six months. All observed changes were found to be within the accuracy of the spectroreflectometer, which is nominally one to two percent. Table 1 qualitatively summarizes some properties of these coatings.

## III. Models of Canopy Reflectance

Investigations into the attenuation of light in a plant canopy were initiated utilizing a one parameter relation customarily referred to as the Bouguer-Lambert law [17]. This approximation accounts satisfactorily for transmittance, while ignoring the reflectance of the medium. Stokes [18] extended the method to account for reflectance by including an additional parameter. He verified his approach in experiments with glass plates. Some years later Schuster [19] reassessed the problem of radiation diffusion and wrote down a system of two equations for radiation transfer in a medium with sources. He assumed that light passing through an infinitesmal layer (*dx*) is increased by the backscattered component and decreased by the forward component. For simplicity he assumed further that the forward (*t*) and back (*s*) scattered components here equal, viz:

$$\begin{array}{l} \frac{dt}{d\psi }=-\mu t-\frac{1}{2}St+\frac{1}{2}Ss,\\ \frac{ds}{dx}=-\mu s-\frac{1}{2}Ss+\frac{1}{2}St, \end{array}$$

where *µ* and *S* are the absorption coefficients. The source terms have been omitted. Rayleigh [20] showed later that equal scattering resulted only for the case where particles are much smaller than the wavelength of light. Silberstein [21] reformulated the Schuster equations eliminating the equality in forward and backscattered components. He also pointed out that the residue of unscattered radiation (*I_x_*) passing through a medium is properly written

$$\frac{dI_{x}}{dx}=-\left(\mu +B+F\right)I_{x},$$

where *B* and *F* represent back and forward scattered components. These results closely resemble those of the Kubelka-Munk [22] representation for canopy scattering,

$$\begin{array}{l} \frac{dI_{x}}{dn}=-\left(\mu +S\right)I_{x}+Ss,\\ \frac{ds}{dn}=\left(\mu +S\right)s-SI_{x}, \end{array}$$

where *I_x_* is now interpreted as the forward scattered radiation.

**Figure 1:**
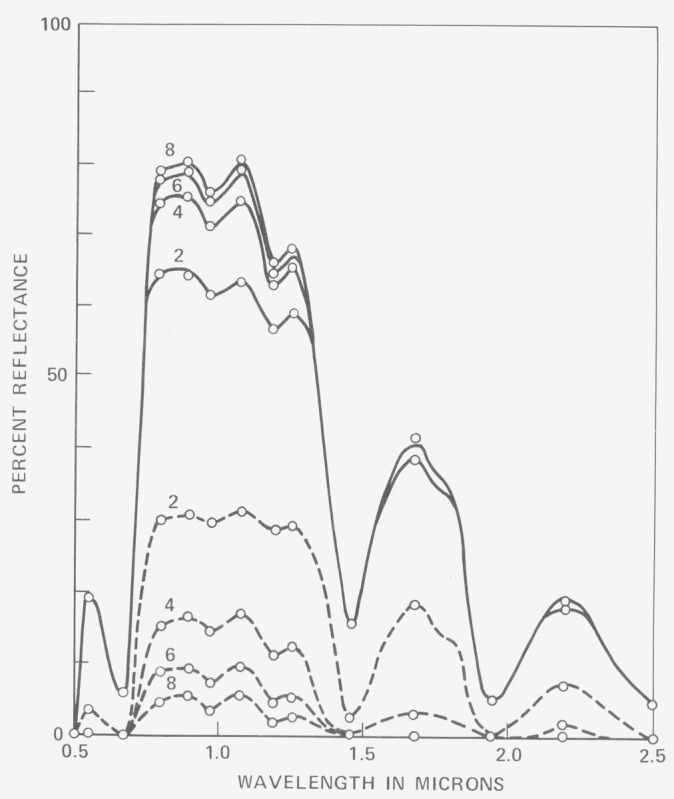
Reflectance (solid lines) and transmittance (dashed lines) of 2, 4, 6, 8, stacked mature cotton leaves. The lines are theoretical; the circles are experimental. Standard deviation between observed and calculated points is about 1 percent [ref. 23].

In this case *t* has been replaced by *I_x_* and *n* represents the cumulative leaf area index.^3^ Figure 1, taken from Allen and Richardson [23] shows some results obtained for stacked cotton leaves. The K–M parameters *µ* and *S* were obtained esperimentally. Duntley [24] combined the equations of Schuster with observation of Silberstein concerning passage of the unscattered beam through a medium. Namely he chose to distinguish the forward diffusing scattered radiation from the unscattered radiation. Under these circumstances three equations result containing six parameters,

$$\begin{array}{l} \frac{dI_{x}}{dx}=-\left(\mu \prime +B\prime +F\prime \right)I_{x},\\ \frac{dt}{dx}=F\prime I_{x}-\mu t-Bt+Fs,\\ -\frac{dS}{dx}=B\prime I_{x}-\mu s-Fs+Bt. \end{array}$$

Primed quantities differentiate the behavior of the unscattered beam (*I_x_*) from that of the diffused portion. First set up to model diffusion in pigmented materials from experiments carried out to give values of the phenomenological constants, it has been subsequently utilized by Suits et. al. [25], to calculate the bidirectional reflectance of corn. The approach necessitates a prior knowledge of the transmittance and reflectance of canopy components, usually only leaves. In addition these parameters are weighted by the projected area on the appropriate component plane. To compute the representation Suits et. al. refer to dividing the canopy into layers, and then rewrite Duntley’s equation for the passage of the unscattered component as

$$\frac{dI}{dx}=kI$$

with *k*=.(*µ*′+*B*′+*F*′) in Duntley’s notation. *I*, *t*, and *S* are now functions of the layer being considered and the position within that layer, *x.* In establishing equations for *k*, *µ*, *B, B*′, and *F*′ plant geometry and optical constants were assessed, and diffusers assumed Lambertian.

**Figure 2:**
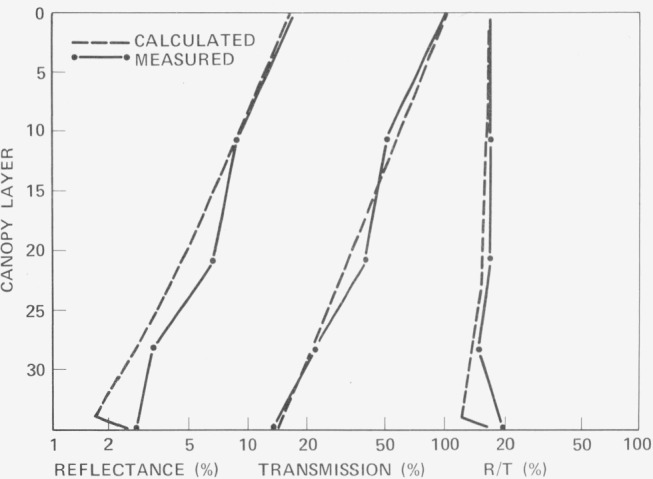
Calculated and measured profiles of reflection R and transmission T in a corn crop. *R* is computed as upward moving radiation divided by incident radiation and *T* as downward moving radiation divided by incident radiation [ref. 29].

Based on the successful application of K–M theory to predict the reflectance and transmittance of stacked cotton leaves, Allen and Richardson [23] indicated that their procedure was sufficiently general for application to plant canopy. Unfortunately this application has not been made by them. Although they do suggest that assuming uniform distributions of leaves would be a reasonable approximation, it was previously shown by Nichiporovich [26] and de Wit [27] not to be valid. Physically, the assumption amounts to suggesting that plant canopy reflectance would not vary during the day. That the effect is present is shown by many authors, in particular by Fritschen [28] for alfalfa, barley, wheat, oats, cotton and sorghum. It is precisely this behavior of crop canopies which led de Wit [27], and Idso and de Wit [29] to develop leaf distribution functions for six leaf inclinations: planophile canopies where horizontal leaves are most frequent; erectophile canopies where vertical leaves are most frequent; plagiophile canopies where leaves assume an oblique inclination; extremophile canopies, where leaves are least frequently at oblique inclinations; spherical canopies where the relative frequencies of inclinations is the same as the relative frequency of the surface elements of a sphere; and a measured leaf distribution for corn. For light incident at *θ*, averaged over the crop as a whole *OP*(*π*/2*–θ*), corrected for the projection of the soil in the direction (*π*/2–*θ*) and weighted for the area per leafy layer, *S*, Idso and de Wit derive for the reflectance *ρ*,

$$\rho ={\left[1-s\frac{OP\left(\pi /2-\theta \right)}{\cos\;\theta }\right]}^{\frac{LAI}{S}}$$

for a leaf area index, LAI, Figure 2 [29] shows a comparison of calculated and measured results for a stand of corn. Agreement appears to be best for the uppermost clusters of leaves. The model of Smith and Oliver [30, 31, 32] treats the interaction of radiant flux with the vegetation canopy utilizing a probability for the distribution of gaps and foliage elements within the canopy. Their starting point is then the Idso-de Wit equation given above for gap probability with

$$\frac{LAI}{n}$$

replaced by the difference between the number of canopy layers *n* and the number of contacts, *k* such that the probability distribution for *k* foliage contacts in the direction *θ_r_* is [31]

$$P\left(k\right)=\left(\frac{n}{k}\right){\left[s\frac{OP\left(\theta_{r}\right)}{\cos\;\left(\theta_{r}\right)}\right]}^{k}{\left[1-s\frac{OP{\left(\theta \right)}_{r}}{\cos\;\left(\theta_{r}\right)}\right]}^{n-k}.$$

**Figure 3:**
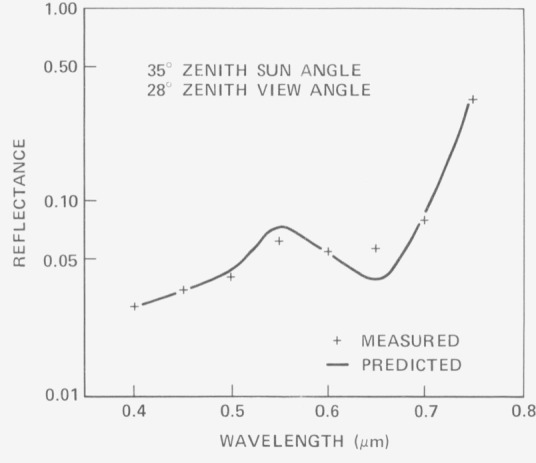
Comparison between model predictions and field observations for a dense canopy of blue grama.

**Figure 4:**
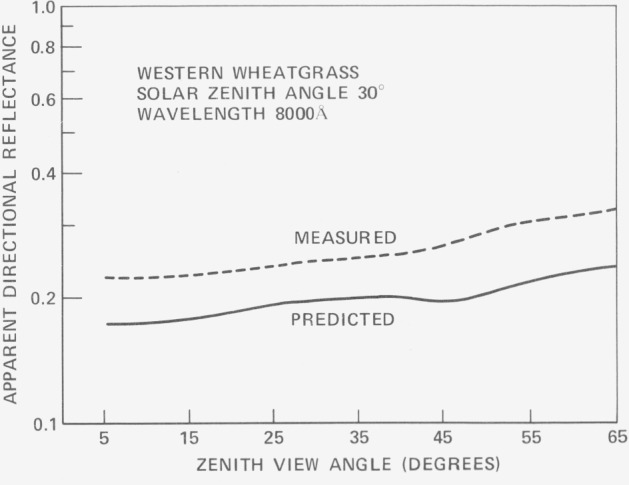
Comparison between model predictions and field observations for a sparse canopy of western wheatgrass. The measurement was made at an azimuth of 45° from the sun and is believed to account for the apparent differences [ref. 31].

**Figure 5:**
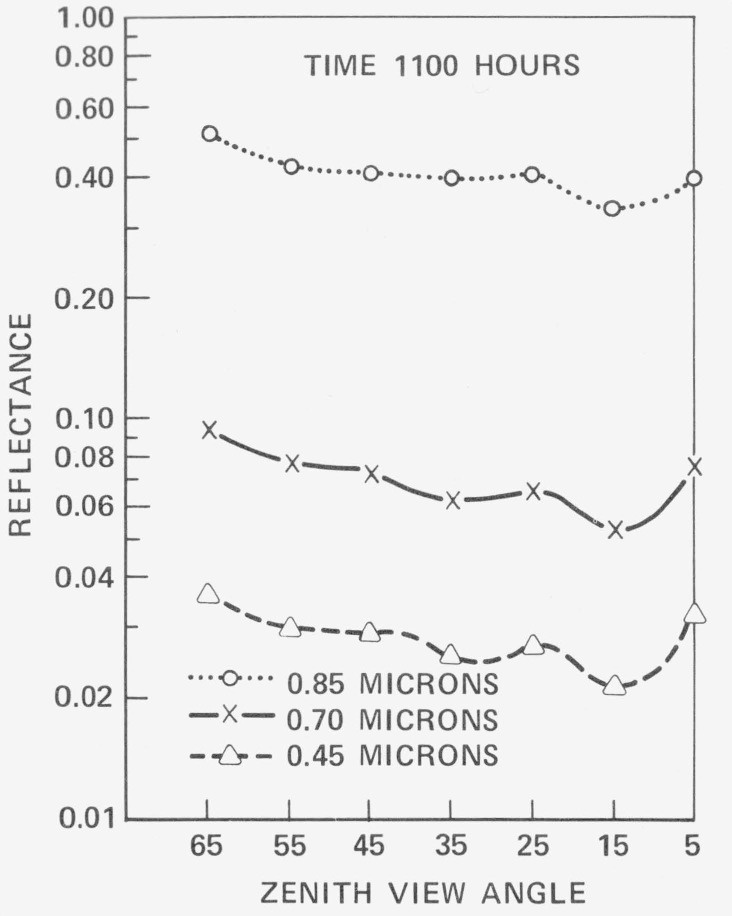
Angular variation in spectral response for three selected wavelengths. The non-Lambertian spatial response for the apparent directional reflectance for the canopy is clearly evident. The solar disk was contained in the 25 degree sensor view angle band [ref. 30].

**Figure 6:**
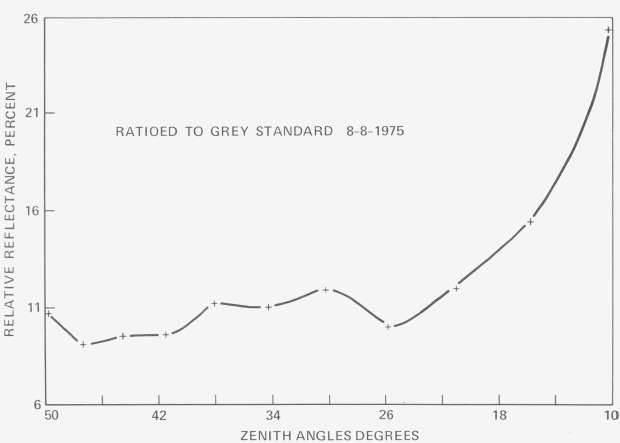
Reflectance of unstressed mature soybean plants taken for LANDSAT band 4 (500–600 nm bandwidth) [ref. 33].

Foliage elements are assumed to act as Lambertian-response surfaces. The problem is the determination of *OP*(*θ_r_*). Idso and de Wit have expressed this quantity in terms of four angles: sun zenith angle, leaf inclination, and two boundary angles differentiating upperside and underside illumination of the leaves. Methods for obtaining an average plant and canopy scattering function have been reviewed by Oliver [31]. Techniques fall into two categories. The first is by a direct mensuration process and the second carried out photographically. One such approach involves the determination of plant geometry from photographs taken for orthogonal planes which are subsequently processed onto a grid. A second approach utilizes infrared photography. A diffuse image of the subject is obtained for bidirectional views. Utilizing these data, Oliver presents numerical methods by which gap and contact probabilities can be obtained. Employing the above equation for *θ_r_*, and applying sequentially through the canopy for all *k* and *n*, there results an expression for the reflectivity *ρ*(*θ′_r_*) [31]:

$$\rho \left(\theta \prime_{r}\right)=\frac{P\left(\theta \prime_{r}\right)}{\sin^{2}\left(\theta_{r}+\Delta \theta_{r}\right)-\sin^{2}\left(\theta \prime_{r}-\Delta \theta_{r}\right)}$$

for incidence in zone (*θ_r_* + Δ*θ_r_*). Figures 3, 4, and 5 show verification results for this model. Each series of curves shows maximum disagreement at about 650 nm. The authors attribute this behavior to possible specular reflection at the long wavelength chlorophyll absorption band. Overall agreement is quite good. In figure 6 relative reflectance is plotted as a function of zenith angle from data obtained by Watlington et. ah, LANDSAT band 4 (500–600 nm). These reflectance data, plotted on a linear scale, show considerably less variation. Because the sensor viewed about one square foot of projected plant area, it is tentatively assumed that the dip at 64° results from polarization of the light. The sharp rise in reflectance beyond this point is as a result attributed to depolarization as the sensor viewing angle increases away from the Brewster Angle.

## IV. Discussion

Based upon the coatings systems mentioned previously, it is apparent they share several formulating concepts to varying degrees. First of all, each pigment was chosen from combinations of elements in the Periodic Table whose electronegativity differences are closely maximized. This being the case, it is hardly possible to extend the ultraviolet absorption cutoff. Creating diffusers from such materials as sapphire, lithuim flouride, magnesium fluoride and fused silica provides little help because roughening a surface or grinding a single high purity crystal simply serves to retract the ultraviolet absorption cutoff. Second, the pigments used are all of the highest purity. Third, the pigment to binder ratio is made as great as possible; for sodium chloride it is infinite.

In connection with the transmission diffuser previously mentioned, the ability of the barium sulphate polyvinyl alcohol system to diffuse a specular beam during a single pass through the coating was unsatisfactory. For this application a thickness of about six mils is normally applied. In a closed system such as an integrating sphere, this problem is not so serious. The black dot artifice of Krueger to block light having passed through the diffuser but once suggests that the diffuser could be more efficient or else its ability to diffuse is intensity dependent. It was pointed out to be by Heath, [34] that the accuracy of the BUV is currently limited solely by fact that the specular component from the diffuser can be measured to no better than 2 percent. Common to crop reflectance models is the assumption that single reflections result in the total diffusion of specular inputs. So unless a canopy can approximate the action of an integrating sphere, the canopies will provide an observer with specular reflections. Then as the spatial resolution is decreased by observing larger and larger canopies, Lambertian behavior will provide an exact description.

It is interesting to note that accurate descriptions of diffusers are difficult to come by because particle shapes, sizes, and their dispersions within binders are not readily amenable to modeling. On the other hand, modeling a canopy for the spacecraft view is a tractable problem, if only for the fact that the components can be directly measured, photographed, and therefore represented geometrically.

Finally, it is interesting to note that the Idso-de Wit canopy model does work for the uppermost vegetation layers by considering geometrical factors only. Absent from the model are provisions for including optical properties. This leads to the conclusion that in setting up leaf distribution functions, probably all specimens were in the nearly turgid state. Subsequently for verification of their model, they chose a crop stand in very nearly the same state. This observation suggests that the evolution of crop canopy geometry with respect to moisture content may serve as a new basis for canopy reflectance modeling.

In conclusion it should be pointed out that in the development image processing algorithms, goniometric data may serve only a tutorial function, unless simpler schemes are developed for its acquisition. In conjunction with the profusion spacecraft imagery now available, however, it could serve the important function of allowing like subjects imaged at different times and in widely spaced locales to be accurately referenced with respect to one another.

## Figures and Tables

**Table 1:** Summary of important properties of coatings Systems employed as diffuse standards

Average reflectance values							
Material	Binder	0.2–0.25 *µ*m	0.25–0.3 *µ*m	0.3–0.8 *µ*m	0.8–2.4 *µ*m	Atmospheric stability	Directional reflectance

Freshly smoked MgO	Electrostatic, water	0. 9	0.98	0. 98	0. 95	Poor	Lambertian to 45 degrees
NaCl	NaCl	Poor	Less than 0.7	. 96	. 96	Poor if RH is greater than 0.85	Unknown
BaSO_4_	PVA	. 85	.93	. 95	. 92	Excellent	Ref. [11]
BaSO_4_	K_2_SO_4_	. 94	.97	. 97	. 92	Excellent	Unknown

## Footnotes

1 Figures in brackets indicate the literature references at the end of this paper.

2 Although not explicitly included, the importance of albedo in weighting solar irradiance is self-evident because the sun is virtually always the dominant heat source by orders of mangnitude.

3 Cumulative leaf area index is defined as the one sided leaf area per unit ground area, and represents the sum over all leaves encountered between top and base of the plant.
